# Cramer-Rao Bounds and Coherence Performance Analysis for Next Generation Radar with Pulse Trains

**DOI:** 10.3390/s130405347

**Published:** 2013-04-22

**Authors:** Xiaowei Tang, Jun Tang, Qian He, Shuang Wan, Bo Tang, Peilin Sun, Ning Zhang

**Affiliations:** 1 Department of Electronic Engineering, Tsinghua University, Beijing 100084, China; E-Mails: tangj_ee@mail.tsinghua.edu.cn (J.T.); wanshuang@mail.tsinghua.edu.cn (S.W.); spl09@mails.tsinghua.edu.cn (P.S.); z315n@126.com (N.Z.); 2 Department of Electronic Engineering, University of Electronic Science and Technology of China, Chengdu 611731, China; E-Mail: qianhe@uestc.edu.cn; 3 Electronic Engineering Institute, Hefei 230037, China; E-Mail: tangbo06@gmail.com

**Keywords:** next generation radar (NGR), Cramer-Rao bound (CRB), Fisher information matrix (FIM), pulse trains, parameter estimation, coherence performance

## Abstract

We study the Cramer-Rao bounds of parameter estimation and coherence performance for the next generation radar (NGR). In order to enhance the performance of NGR, the signal model of NGR with master-slave architecture based on a single pulse is extended to the case of pulse trains, in which multiple pulses are emitted from all sensors and then integrated spatially and temporally in a unique master sensor. For the MIMO mode of NGR where orthogonal waveforms are emitted, we derive the closed-form Cramer-Rao bound (CRB) for the estimates of generalized coherence parameters (GCPs), including the time delay differences, total phase differences and Doppler frequencies with respect to different sensors. For the coherent mode of NGR where the coherent waveforms are emitted after pre-compensation using the estimates of GCPs, we develop a performance bound of signal-to-noise ratio (SNR) gain for NGR based on the aforementioned CRBs, taking all the estimation errors into consideration. It is shown that greatly improved estimation accuracy and coherence performance can be obtained with pulse trains employed in NGR. Numerical examples demonstrate the validity of the theoretical results.

## 1. Introduction

Large aperture high-power phased array radar has played an important role in long-range surveillance, tracking and discrimination, owing to its capability of obtaining high signal-to-noise ratio (SNR) echoes. Typical such radars include the USA's Ground Based Radar-Prototype (GBR-P) and the Sea-Based X-Band (SBX) radar [1]. However, large size and heavy weight usually make them difficult to transport and deploy and hence, easy to be attacked in practice. In order to achieve high SNR gain while maintaining acceptable sensor size, a novel radar architecture has recently been proposed by the Lincoln Laboratory, *i.e.*, the next generation radar (NGR) [2], where the large aperture phased array radar is made up of several transportable distributed sub-apertures or sub-radars. It is shown that NGR has improved mobility, stronger survival ability and similar processing gain compared with the traditional large aperture phased array radar. Moreover, an experimental NGR system with two radars has been constructed by the Lincoln Laboratory, which is reported in [3] to have obtained inspiring coherent processing gain in field tests, showing its good application prospects.

In this paper, we consider NGR with a master-slave architecture, where all the radars transmit signals and only the master radar receives the echoes. It is known that the maximum echo power can be achieved only when we make all the transmitted signals arrive at the target at the same time and in-phase, namely, the coherence gain is obtained via coherent processing. However, the distributed architecture of NGR makes it difficult to coherently combine signals for two reasons. First, the range from a target to different radars may be different, leading to echoes with different propagation time delays and phases; second, each radar has an independent local oscillator with different transmit and receive (T/R) phases, which also adds phase shifts to echoes. Since both the T/R phases and the phase caused by propagation delay can influence the coherence gain, we add the two phases together and name the sum as total phase. In NGR, both the mismatches of time and phase can cause performance degradation. To overcome this shortage, an operation procedure of two steps has been proposed [2]. In the first step which is also called the MIMO mode, each radar transmits a probing signal (usually orthogonal waveforms) to estimate the *time delay differences* and *total phase differences* between sub-radars and the master radar, and they are referred to as coherence parameters (CPs) [2–5]. In the second step which is also called the coherent mode, all radars transmit coherent waveforms adjusted by the estimated CPs from MIMO mode. Clearly, the estimation accuracy of CPs greatly impacts the coherence gain that can be obtained by NGR, which raises two important questions: What is the best estimation accuracy for the CPs? How much coherence gain can we get assuming that estimation accuracy is achievable?

Another problem in NGR lies in the constraint of system size, *i.e.*, the number of radars cannot be arbitrarily large in practice. Thus, the maximum SNR gain that can be obtained merely through the spatially coherent processing of distributed radars is limited, which is unfavorable in detecting and tracking long-range weak targets. To settle this problem, it is natural and essential to emit pulse trains in NGR, which means we will accumulate the energy of echoes not only from different radars but also from multiple pulses. In NGR transmitting pulse trains, new questions immediately emerge: How will the introduction of pulse trains affect the estimation accuracy of aforementioned CPs? Are there any new parameters that need to be estimated? If any, what is the best estimation accuracy for those parameters? What is the optimal coherence performance for NGR with pulse trains?

A thorough review of the existing literature on NGR reveals that the present signal models in NGR are all based on single pulse schemes [2–5], whereas the transmission of pulse trains has not been considered yet. From the aspect of parameter estimation, [4] derived the CRBs of time delay differences and T/R phase differences for a general NGR architecture, but the CRBs of total phase differences are not given. Therefore, the CRBs of CPs have not been thoroughly worked out so far, according to the definition of CPs. In the field of performance analysis, [4] derived the performance bound of NGR based only on the CRBs of T/R phase differences, assuming that all time delay differences are ideally compensated. In [5] a formula of coherence gain taking all types of estimation errors into consideration was presented, but the performance bounds were not analyzed. Therefore, the performance bound analysis of NGR still remains an unresolved problem.

In addition, it is worth pointing out that the parameter estimation of NGR should be distinguished from the parameter estimation of MIMO radar which has been studied in [6–17], despite their superficial similarities in emitting orthogonal waveforms. Their differences are: first, the central parameters of concern in NGR and MIMO radar are different. MIMO radars are focused on target localization accuracy, *i.e.*, the x-y coordinate or the x-y velocity of a target, while NGR cares about time delay differences and total phase differences with respect to the target, *i.e.*, the CPs. Second, the parameters of phases are modeled and treated differently in NGR and MIMO radar. In MIMO radar, phase synchronization errors are modeled as random variables which are used to evaluate the *average* performance degradation [14–16], and they need not to be estimated, thus their CRBs are of no interest, while in NGR the parameters of phases are modeled as deterministic unknowns that need to be estimated for compensation so their CRBs are of high concern.

In this paper, we make the following contributions which also answer the questions at the end of paragraphs two and three. All the contributions below are useful and instructive for the system design and performance analysis of NGR:

(a) The NGR signal model based on a single pulse is extended to the case of pulse trains for the first time, and the concept of spatial coherence is extended to joint space-time coherence for NGR. The extension to pulse trains benefits the detection and tracking of weak targets and helps control the system scale of NGR.
(b) The original coherence parameters (CPs) of NGR are extended to the generalized coherence parameters (GCPs), with *Doppler frequencies* involved. Since target echoes coming from different radars usually have different Doppler frequencies, they must also be estimated and compensated. The extension to GCPs is essential in characterizing the multi-pulse model in (a).
(c) The closed-form CRBs of the GCPs are derived based on the signal model in (a), and verified through simulations, thus providing a lower bound for the estimation accuracy of the GCPs and a criterion for the performance evaluation of different estimation algorithms.
(d) The formula of coherence gain for NGR is derived and the performance bound is analyzed based on the CRBs in (c) with all types of estimation errors considered, thus providing an upper bound for the SNR gain performance of NGR.

The paper is organized as follows: in Section 2, we present the NGR signal model with pulse trains and specifically define the GCPs. In Section 3, we derive the CRB for parameter estimation. In Section 4, we present the analytical formula of coherence performance. Simulation results and discussions are shown in Section 5, and Section 6 concludes the paper.

## 2. System Model and Parameter Definitions

**Figure 1.**
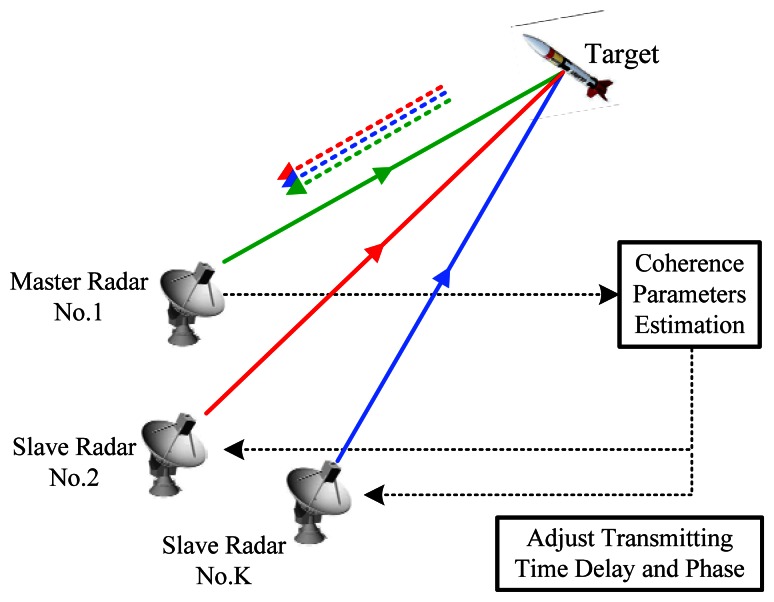
The master-slave architecture of NGR.

The system model of NGR with master-slave architecture is illustrated in Figure 1. Without loss of generality, we assume that there are *K* radars with Radar No.1 being the master radar.

The pulse signal transmitted by the *k*th transmitter is:

(1) $$s_{k}(t)e^{j2\pi f_{c}t+j\theta_{k}^{t}},k=1,\cdots ,K$$

where *s_k_*(*t*) is the baseband signal of the *k*th transmitter, *f*_c_ is the carrier frequency, and $\theta_{k}^{t}$ represents the phase of the local oscillator at the *k*th transmitter. We define the effective bandwidth of *s_k_*(*t*) as:

(2) $$\beta_{k}=\sqrt{\int f^{2}{\left|S_{k}(f)\right|}^{2}df/\int {\left|S_{k}(f)\right|}^{2}df}$$

where *S_k_*(*f*) is the Fourier transform of *s_k_*(*t*). Further, we assume that the effective bandwidths of all the signals are equal, *i.e.*, *β* = *β_k_*, *k* = 1,…, *K*.

As mentioned above, we consider the case of transmitting multiple pulses. Assume that *N* pluses are transmitted consecutively and reflected by a moving point target. For convenience, assume that the range-rates of the target with respect to all radars remain constant and the target does not move across a range cell within the observation interval, which implies that no range migration occurs. The range from the target to the *k*th transmitter when transmitting the *n*th pulse can be expressed as:

(3) $$\begin{array}{cc} R_{k,n}=R_{k,0}-nT•{\dot{R}}_{k}, & n=0,1,\cdots ,N-1 \end{array}$$

where *R_k_*_,0_ is the initial range from the target to the *k*th transmitter, *T* is the pulse repetition interval (PRI), and *Ṙ*_k_ is the range-rate of the target with respect to the *k*th transmitter.

Then, the propagation time of the *n*th pulse in the *k*th path, *i.e.*, the path from the *k*th transmitter to the first receiver (*i.e.*, the master radar), can be expressed as:

(4) $$\begin{array}{cc} \tau_{1k,n}=\tau_{1k,0}-nT•\frac{{\dot{R}}_{1k}}{c}, & n=0,1,\cdots ,N-1 \end{array}$$

where *c* represents the speed of light, *τ*_1_*_k_*_,0_ = (*R*_1,0_ + *R_k_*_,0_)/*c* and *Ṙ*_1_*_k_* = *Ṙ*_1_ + *Ṙ*_k_ represent the propagation time of the first pulse and the range-rate of the *k*th path, respectively.

For simplicity, we assume that the target is non-scintillating, *i.e.*, its complex reflection coefficient is identical for each propagation path, and is denoted as ξ̄ The *n*th pulse echo received at the master radar is a superposition of the returns contributed by the emitted signals from *K* transmitters:

(5) $$\sum_{k=1}^{K}\bar{\xi }s_{k}(t-\tau_{1k,n})e^{j2\pi f_{c}(t-\tau_{1k,n})+j\theta_{k}^{t}}$$

The received signal in Equation (5) is then mixed up with the master radar's local oscillator $e^{-j2\pi f_{c}t+j\theta_{1}^{r}}$ and down-converted to base-band, where *θr* 1represents the phase of the first receiver. Substituting Equation (4) into (5), the signal after down-conversion can be expressed as:

(6) $$\begin{array}{ll} r_{1,n}(t) & =\sum_{k=1}^{K}\bar{\xi }s_{k}(t-\tau_{1k,n})e^{-j2\pi f_{c}\tau_{1k,n}+j\theta_{k}^{t}+j\theta_{1}^{r}}+w_{1,n}(t)\\ & =\sum_{k=1}^{K}\bar{\xi }s_{k}(t-\tau_{1k,n})e^{-j2\pi f_{c}\left(\tau_{1k,0}-nT•\frac{{\dot{R}}_{1k}}{c}\right)+j\theta_{k}^{t}+j\theta_{1}^{r}}+w_{1,n}(t) \end{array}$$

where *w*_1_,*_n_*(*t*) is the noise of the first receiver while receiving the *n*th pulse. We assume that *w*_1_,*_n_*(*t*) is a temporally and spatially white zero-mean complex Gaussian random process. More specifically, we have:

(7) $$\text{E}\left\{w_{1,n}(t)w_{1,m}^{*}(u)\right\}=\sigma_{w}^{2}\delta \left(t-u\right)\delta \left(m-n\right)$$

where $\sigma_{w}^{2}$ is a constant and *δ*(*t*) is the dirac function.

By denoting $\xi =\bar{\xi }e^{-j2\pi f_{c}\tau_{11,0}+j\theta_{1}^{t}+j\theta_{1}^{r}}=\xi_{r}+j\xi_{i}$, $\Delta \theta_{k}^{t}=\theta_{k}^{t}-\theta_{1}^{t}$ and Δ*τ_k_* = *τ*_1_*_k_*_,0_ − *τ*_11,0_, Equation (6) can be written as:

(8) $$\begin{array}{ll} r_{1,n}(t) & =\sum_{k=1}^{K}\xi s_{k}(t-\tau_{1k,n})e^{-j2\pi f_{c}\Delta \tau_{k}+j\Delta \theta_{k}^{t}+j2\pi f_{c}•\frac{{\dot{R}}_{1k}}{c}•nT}+w_{1,n}(t)\\ & =\sum_{k=1}^{K}\xi s_{k}(t-\tau_{1k,n})e^{j\Delta \phi_{k}^{t}+j\varphi_{k}•n}+w_{1,n}(t) \end{array}$$

where $\Delta \theta_{k}^{t}$ represents the *T/R phase difference* between the *k*th transmitter and the first transmitter, $\Delta \phi_{k}^{t}=-2\pi f_{c}\Delta \tau_{k}+\Delta \theta_{k}^{t}$ represents the *total phase difference* between the *k*th path and the first path and $\varphi_{k}=2\pi f_{c}T\overset{•}{R_{1k}}/c$ represents the normalized *Doppler frequency* of the *k*th path. Applying the previous assumption that no range migration occurs during the observation interval, we have *s_k_*(*t* − *τ*_1_*_k_*,*_n_*) ≈ *s_k_*(*t* − *τ*_1_*_k_*_,0_) approximately, and Equation (8) can be written as:

(9) $$\begin{array}{ll} r_{1,n}(t) & =\sum_{k=1}^{K}\xi s_{k}(t-\tau_{k})e^{j\Delta \phi_{k}^{t}+j\varphi_{k}•n}+w_{1,n}(t)\\ & =\sum_{k=1}^{K}\xi s_{k}(t-\tau_{1}-\Delta \tau_{k})e^{j\Delta \phi_{k}^{t}+j\varphi_{k}•n}+w_{1,n}(t) \end{array}$$

where we replace *τ*_1_*_k_*_,0_ with *τ_k_* here and hereinafter for simplicity and Δ*τ_k_* = *τ_k_* − *τ*_1_ represents the *time delay differences* between the *k*th path and the first path.

It is obvious that $\Delta \tau_{1}=\Delta \theta_{1}^{t}=0$ and $\Delta \phi_{1}^{t}=-2\pi f_{c}\Delta \tau_{1}+\Delta \theta_{1}^{t}=0$, which do not need to be estimated. From Equation (9) we define a parameter vector consisting of deterministic unknowns as:

(10) $$\upsilon ={\left[\Delta \tau^{T},\Delta \psi^{T},\varphi^{T},\xi_{r},\xi_{i}\right]}^{T}$$

where Δ***τ*** ∈ ℝ^(k-1)×1^, Δ**ψ** ∈ ℝ^(k-1)×1^, **φ** ∈ ℝ^(k-1)×1^ with their expressions as:

(11) $$\Delta \tau ={\left[\Delta \tau_{2},\Delta \tau_{3},\cdots ,\Delta \tau_{K}\right]}^{T}$$

(12) $$\Delta \psi ={\left[\Delta \phi_{2}^{t},\Delta \phi_{3}^{t},\cdots ,\Delta \phi_{K}^{t}\right]}^{T}$$

(13) $$\varphi ={\left[\varphi_{1},\varphi_{2},\cdots ,\varphi_{K}\right]}^{T}$$

Note that the *time delay differences* in Equation (11), *total phase differences* in Equation (12) and *Doppler frequencies* in Equation (13) are the GCPs as defined in Section 1.

## 3. CRBs for Parameter Estimation

In this section, we derive the CRBs of the GCPs. The Fisher information matrix (FIM) is calculated first. Then the CRBs are obtained by inverting FIM [18]. Finally, some remarks are given.

### 3.1. FIM of Intermediate Parameters

We find it difficult to compute the FIM of the GCPs directly, thus an intermediate parameter vector **θ** is introduced:

(14) $$\theta ={\left[\tau^{T},\Delta \psi^{T},\varphi^{T},\xi_{r},\xi_{i}\right]}^{T}$$

where ***τ*** = [*τ*_1_, *τ*_2_, ⋯, *τ*_k_]*^T^* ∈ ℝ^K×1^. Next we compute the FIM of **θ**, which is denoted as **I**(**θ**), to further obtain the CRBs of GCPs.

As the noise components in each pulse is independent and identically distributed (i.i.d.) Gaussian, the log-likelihood function of the received signal given in Equation (9) can be expressed as:

(15) $$\log p(r;\theta )=-\frac{1}{\sigma_{w}^{2}}\sum_{n=0}^{N-1}\int_{T}{\left|r_{1,n}(t)-\sum_{k=1}^{K}\xi s_{k}(t-\tau_{k})e^{j\Delta \phi_{k}^{t}+j\varphi_{k}•n}\right|}^{2}dt$$

where ***r*** = [*r*_1,0_(*t*),*r*_1,1_(*t*), ⋯, *r*_1_,*_N_*_-1_(*t*)]*^T^*, and 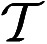 denotes the observation time in a PRI.

The computation of **I**(**θ**) is provided in Appendix A and the final result is:

(16) $$I(\theta )=\left[\begin{array}{cc} T_{K\times K} & 0_{K\times (2K+1)}\\ 0_{(2K+1)\times K} & G_{\left(2K+1)\times (2K+1\right)} \end{array}\right]$$

where:

(17) $$\text{T}=\frac{1}{\sigma_{w}^{2}}{\left|\xi \right|}^{2}8\pi^{2}\beta^{2}N{\text{I}}_{K}$$

(18) $$G=\frac{2}{\sigma_{w}^{2}}\left[\begin{array}{ccc} A & D & E\\ D^{T} & B & F\\ E^{T} & F^{T} & C \end{array}\right]$$

(19) $$A={\left|\xi \right|}^{2}N{\text{I}}_{K-1}$$

(20) $$B={\left|\xi \right|}^{2}\frac{N\left(N-1\right)\left(2N-1\right)}{6}{\text{I}}_{K}$$

(21) $$C=KNI_{2}$$

(22) $$D={\left|\xi \right|}^{2}\left[\begin{array}{cc} 0_{(K-1)\times 1} & \frac{N\left(N-1\right)}{2}{\text{I}}_{K-1} \end{array}\right]$$

(23) $$E=N\left[\begin{array}{cc} 1_{(K-1)\times 1} & 1_{(K-1)\times 1} \end{array}\right]\;\left[\begin{array}{cc} -\xi_{i} & \\ & \xi_{r} \end{array}\right]$$

(24) $$F=\frac{N\left(N-1\right)}{2}\left[\begin{array}{cc} 1_{K\times 1} & 1_{K\times 1} \end{array}\right]\;\left[\begin{array}{cc} -\xi_{i} & \\ & \xi_{r} \end{array}\right]$$

where **I***_K_* represents the *K* × *K* identity matrix, **0***_p_*_×_*_q_* and **1***_p_*_×_*_q_* represent a *p* × *q* zero matrix and a *p* × *q* matrix with all elements being unit, respectively. We denote **1***_p_*_×_*_q_* as **J***_p_* when *p* = *q*. Note that **T**, **A**, **B** and **C** contain the second-order derivatives with respect to **τ**, Δ**ψ**, **φ** and [*ξ_r_*, *ξ_i_*]*^T^*, respectively, **D** contains the second-order derivatives with respect to both Δ**ψ** and **φ**, **E** contains the second-order derivatives with respect to both Δ**ψ** and [*ξ_r_*, *ξ_i_*]*^T^*, and **F** contains the second-order derivatives with respect to both **φ** and [*ξ_r_*, *ξ_i_*]*^T^*. For convenience, we define the CRB matrix (CRBM) as the inverse matrix of FIM and denote CRBM by **CRB_θ_**, *i.e.*, **CRB_θ_**= **I**^−1^(**θ**).

### 3.2. CRB of Time Delay Differences

The CRBM of **τ** can be obtained from Equations (16) and (17):

(25) $$\begin{array}{ll} CRB_{\tau } & =T^{-1}\\ & =\frac{\sigma_{w}^{2}}{8\pi^{2}\beta^{2}N{\left|\xi \right|}^{2}}{\text{I}}_{K}\\ & =\frac{1}{8\pi^{2}\beta^{2}\text{NSN}R_{in}}{\text{I}}_{K} \end{array}$$

where $SNR_{in}={\left|\xi \right|}^{2}/\sigma_{w}^{2}$ is defined as the input SNR at the receiver.

Next we apply the chain rule of CRB to compute the CRBM of Δ**τ** by using the relationship between Δ**τ** and **τ**:

(26) $$\Delta \tau_{k}=\tau_{k}-\tau_{1},k=2,\cdots ,K$$

which gives a Jacobian matrix as:

(27) $$\frac{\partial g(\tau )}{\partial \tau }=\left[\begin{array}{cc} -1_{(K-1)\times 1} & {\text{I}}_{K-1} \end{array}\right]$$

where **g**(**τ**) = Δ**τ**. Then the CRBM of Δ**τ** is:

(28) $$\begin{array}{ll} CRB_{\Delta \tau } & =\frac{\partial g(\tau )}{\partial \tau }CRB_{\tau }{\left(\frac{\partial g(\tau )}{\partial \tau }\right)}^{T}\\ & =\frac{1}{8\pi^{2}\beta^{2}\text{NSN}R_{in}}\left[\begin{array}{cc} -1_{(K-1)\times 1} & {\text{I}}_{K-1} \end{array}\right]{\text{I}}_{K}\left[\begin{array}{c} -1_{(K-1)\times 1}^{T}\\ {\text{I}}_{K-1} \end{array}\right]\\ & =\frac{{\text{I}}_{K-1}+{\text{J}}_{K-1}}{8\pi^{2}\beta^{2}\text{NSN}R_{in}} \end{array}$$

Extracting the diagonal elements of **CRB**_Δ_**_τ_**, the CRBs of time delay differences are expressed as:

(29) $$CRB_{\Delta \tau_{k}}=\frac{1}{N}\frac{1}{SNR_{in}}\frac{1}{4\pi^{2}\beta^{2}},k=2,\cdots ,K$$

which coincides with the result in [4] for the case of *K* transmitters and one receiver, except that a coefficient of 1/*N* is multiplied, indicating the improvement on estimation accuracy by pulse integration.

### 3.3. CRB of Total Phase Differences and Doppler Frequencies

For clarity, we rewrite matrix **G** as:

(30) $$G=\frac{2}{\sigma_{w}^{2}}\left[\begin{array}{cc} \Sigma & U\\ V & C \end{array}\right]$$

where:

(31) $$\Sigma =\left[\begin{array}{cc} A & D\\ D^{T} & B \end{array}\right]$$

(32) $$U=\left[\begin{array}{c} E\\ F \end{array}\right]$$

(33) $$V=U^{T}$$

Using the matrix inversion lemma [19], the inverse matrix of **G**, which corresponds to the CRBM of Δ**ψ**, **φ** and [*ξ_r_*, *ξ_i_*]*^T^*, can be expressed as:

(34) $$\begin{array}{ll} G^{-1} & =\frac{\sigma_{w}^{2}}{2}{\left[\begin{array}{cc} \Sigma & U\\ V & C \end{array}\right]}^{-1}\\ & =\frac{\sigma_{w}^{2}}{2}\left[\begin{array}{cc} {\left(\Sigma -UC^{-1}V\right)}^{-1} & -\Sigma^{-1}U{\left(C-V\Sigma^{-1}U\right)}^{-1}\\ -C^{-1}V{\left(\Sigma -UC^{-1}V\right)}^{-1} & {\left(C-V\Sigma^{-1}U\right)}^{-1} \end{array}\right] \end{array}$$

where the submatrix (**Σ** − **UC**^−1^**V**)^−1^ corresponds to the CRBM of Δ**ψ** and **φ** which are of interest and (**C** − **VΣ**^−1^**U**)^−1^ corresponds to the CRBM of [*ξ_r_*, *ξ_i_*]*^T^* which are nuisance.

From Equations (21) and (31–33), we have:

(35) $$\begin{array}{ll} \Sigma -UC^{-1}V & =\left[\begin{array}{cc} A & D\\ D^{T} & B \end{array}\right]-\frac{1}{KN}\left[\begin{array}{c} E\\ F \end{array}\right]\;\left[\begin{array}{cc} E^{T} & F^{T} \end{array}\right]\\ & =\left[\begin{array}{cc} A^{\prime } & U^{\prime }\\ V^{\prime } & D^{\prime } \end{array}\right] \end{array}$$

where:

(36) $$A^{\prime }=A-\frac{1}{KN}EE^{T}={\left|\xi \right|}^{2}N\left({\text{I}}_{K-1}-\frac{1}{K}{\text{J}}_{K-1}\right)$$

(37) $$U^{\prime }=D-\frac{1}{KN}EF^{T}={\left|\xi \right|}^{2}\frac{N\left(N-1\right)}{2}\left[\begin{array}{cc} -\frac{1}{K}1_{(K-1)\times 1} & {\text{I}}_{K-1}-\frac{1}{K}{\text{J}}_{K-1} \end{array}\right]$$

(38) $$D^{\prime }=B-\frac{1}{KN}FF^{T}={\left|\xi \right|}^{2}\frac{N\left(N-1\right)\left(2N-1\right)}{6}\left[{\text{I}}_{K}-\frac{3\left(N-1\right)}{2\left(2N-1\right)K}{\text{J}}_{K}\right]$$

(39) $$V^{\prime }={U^{\prime }}^{T}$$

Then, using the matrix inversion lemma once again, we have:

(40) $$\begin{array}{ll} {\left(\Sigma -UC^{-1}V\right)}^{-1} & ={\left[\begin{array}{cc} A^{\prime } & U^{\prime }\\ V^{\prime } & D^{\prime } \end{array}\right]}^{-1}\\ & =\left[\begin{array}{cc} {\left(A^{\prime }-U^{\prime }{D^{\prime }}^{-1}V^{\prime }\right)}^{-1} & -{A^{\prime }}^{-1}U^{\prime }{\left(D^{\prime }-V^{\prime }{A^{\prime }}^{-1}U^{\prime }\right)}^{-1}\\ -{D^{\prime }}^{-1}V^{\prime }{\left(A^{\prime }-U^{\prime }{D^{\prime }}^{-1}V^{\prime }\right)}^{-1} & {\left(D^{\prime }-V^{\prime }{A^{\prime }}^{-1}U^{\prime }\right)}^{-1} \end{array}\right] \end{array}$$

where (**A'** − **U'D'**^−1^**V'**)^−1^ and (**D'** − **V'A'**^−1^**U'**)^−1^ correspond to the CRBM of Δ**ψ** and **φ**, respectively.

Using the following two equations:

(41) $${\left({\text{I}}_{K}+c{\text{J}}_{K}\right)}^{-1}={\text{I}}_{K}-\frac{c}{1+cK}{\text{J}}_{K}$$

(42) $$\frac{1}{K}{\text{J}}_{K-1}={\text{J}}_{K-1}\left({\text{I}}_{K-1}-\frac{1}{K}{\text{J}}_{K-1}\right)=\left({\text{I}}_{K-1}-\frac{1}{K}{\text{J}}_{K-1}\right){\text{J}}_{K-1}$$

where *c* is a constant and $c\neq -\frac{1}{K}$, we obtain:

(43) $$CRB_{\Delta \psi }=\frac{1}{SNR_{in}}\frac{\left(2N-1\right)}{N\left(N+1\right)}\left({\text{I}}_{K-1}+{\text{J}}_{K-1}\right)$$

(44) $$CRB_{\varphi }=\frac{1}{SNR_{in}}\frac{6}{N\left(N^{2}-1\right)}{\text{I}}_{K}$$

where the definition of *SNR_in_* is the same as in Equation (25).

Finally, the CRBs of total phase differences and Doppler frequencies are expressed as:

(45) $$CRB_{\Delta \phi_{k}^{t}}=\frac{1}{SNR_{in}}\frac{2\left(2N-1\right)}{N\left(N+1\right)},k=2,\cdots ,K$$

and:

(46) $$CRB_{\varphi_{k}}=\frac{1}{SNR_{in}}\frac{6}{N\left(N^{2}-1\right)},k=1,\cdots ,K$$

respectively.

### 3.4. Remarks on CRB Results

Based on Equations (29), (45) and (46), we have the following remarks on the CRB results:

(1) All the CRBs are irrelevant to the number of radars *K*, which is the characteristic of the master-slave architecture. In the estimation procedure of MIMO mode, *K* orthogonal signals are extracted from the mixture echo of the master radar to estimate *K*−1 time delay differences, *K*−1 phase differences and *K* Doppler frequencies. This implies that more radars bring more parameters to be estimated, and the estimation accuracy does not increase as *K* increases.
(2) The CRB of the total phase differences in Equation (45) only depends on input SNR and the pulse number *N*. Note that the CRB of the T/R phases in [4] is proportional to the squared ratio of the carrier frequency to the effective bandwidth (*f*_c_/*β*)^2^. Obviously, the latter is much higher than the former under the assumption of narrowband signals, and performance analysis based merely on the mismatch of T/R phases would be inappropriate and more or less discouraging, as we will see in Section 5.3.
(3) When the pulse number *N* is relatively large, the CRB of Δ*τ_k_*, Δ*Φt k* and *φ_k_* is proportional to 1/*N*, 4/*N* and 6/*N*^3^, respectively. Intuitively, for the estimation of Δ*τ_k_*, the coherent integration of *N* pulses is equivalent to increasing the input SNR *N* times. In contrast, for the estimation of Δ*Φt k*, due to its coupling with the estimation of *φ_k_*, the coherent integration of *N* pulses results in a reduced equivalent SNR gain of *N*/4. The CRB of *φ_k_* descends the fastest among all the GCPs as *N* increases.

## 4. Coherence Performance Analysis

In this section, we analyze the coherence performance of NGR with pulse trains. The signal model in coherent mode is presented first. Then the formula of coherence gain is provided. Finally, some remarks are given.

### 4.1. Signal Model in Coherent Mode

We assume that the GCPs are stable when NGR switches from MIMO mode to coherent mode, so that the estimates for GCPs in MIMO mode can be applied to adjust the phases and time delays on transmit. The estimates for GCPs are defined as:

(47) $$\Delta \hat{\tau }={\left[\Delta {\hat{\tau }}_{2},\Delta {\hat{\tau }}_{3},\cdots ,\Delta {\hat{\tau }}_{K}\right]}^{T}$$

(48) $$\Delta \hat{\psi }={\left[\Delta {\hat{\phi }}_{2}^{t},\Delta {\hat{\phi }}_{3}^{t},\cdots ,\Delta {\hat{\phi }}_{K}^{t}\right]}^{T}$$

(49) $$\hat{\varphi }={\left[{\hat{\varphi }}_{1},{\hat{\varphi }}_{2},\cdots ,{\hat{\varphi }}_{K}\right]}^{T}$$

We use $\delta \tau_{k}=\Delta \tau_{k}-\Delta {\hat{\tau }}_{k}$, $\delta \phi_{k}^{t}=\Delta \phi_{k}^{t}-\Delta {\hat{\phi }}_{k}^{t}$ and $\delta \varphi_{k}=\varphi_{k}-{\hat{\varphi }}_{k}$ to represent the estimation errors. In coherent mode, the *n*th pulse transmitted by the *k*th transmitter can be expressed as:

(50) $$s(t+\Delta {\hat{\tau }}_{k})e^{j2\pi f_{c}t+j\Delta \theta_{k}^{t}-j\Delta {\hat{\phi }}_{k}^{t}-j{\hat{\varphi }}_{k}•n}$$

where *s*(*t*) is the baseband coherent waveform, *s*(*t* + Δ *τ̇*_k_) represents the time delay adjustment, $e^{-j\Delta {\hat{\phi }}_{k}^{t}}$ compensates the total phase difference and *e*^−^*^j^*^φ̂^_k_^•n^ compensates the phase caused by Doppler.

From Equations (8), (9) and (50), the *n*th pulse echo received by the master radar after propagation and down-conversion is:

(51) $$\begin{array}{ll} r_{1,n}(t) & =\sum_{k=1}^{K}\xi s(t-\tau_{k}+\Delta {\hat{\tau }}_{k})e^{-j2\pi f_{c}\Delta \tau_{k}+j\Delta \theta_{k}^{t}+j\varphi_{k}•n-j\Delta {\hat{\phi }}_{k}^{t}-j{\hat{\varphi }}_{k}•n}+w_{1,n}(t)\\ & =\sum_{k=1}^{K}\xi s(t-\tau_{1}-\Delta \tau_{k}+\Delta {\hat{\tau }}_{k})e^{j\left(\Delta \phi_{k}^{t}-\Delta {\hat{\phi }}_{k}^{t}\right)+j\left(\varphi_{k}-{\hat{\varphi }}_{k}\right)•n}+w_{1,n}(t)\\ & =\sum_{k=1}^{K}\xi s(t-\tau_{1}-\delta \tau_{k})e^{j\delta \phi_{k}^{t}+j\delta \varphi_{k}•n}+w_{1,n}(t) \end{array}$$

Since all the GCPs have been compensated on transmit, the *N* pulses received by the master radar can be directly accumulated to achieve coherent integration, and the final integrated signal is:

(52) $$\begin{array}{ll} r(t) & =\sum_{n=0}^{N-1}r_{1,n}(t)\\ & =\sum_{n=0}^{N-1}\sum_{k=1}^{K}\xi s(t-\tau_{1}-\delta \tau_{k})e^{j\delta \phi_{k}^{t}+j\delta \varphi_{k}•n}+\sum_{n=0}^{N-1}w_{1,n}(t)\\ & =\xi \sum_{k=1}^{K}s(t-\tau_{1}-\delta \tau_{k})e^{j\delta \phi_{k}^{t}}A(\delta \varphi_{k})+\sum_{n=0}^{N-1}w_{1,n}(t) \end{array}$$

where $A(\delta \varphi_{k})=\overset{N-1}{\underset{n=0}{\text{∑}}}e^{j\delta \varphi_{k}•n}$ represents the impact of Doppler estimation errors.

For simplicity, *δτ_k_*, $\delta \phi_{k}^{t}$ and *δφ_k_* are assumed to be independent with Gaussian distribution, *i.e.*, $\delta_{\tau_{k}}\sim N(0,\sigma_{\tau }^{2})$, *k* = 2,…, *K*, $\delta \phi_{k}^{t}\sim N(0,\sigma_{\phi }^{2})$, *k* = 2,…, *K*, $\delta \varphi_{k}\sim N(0,\sigma_{\varphi }^{2})$, *k* = 1,…, *K*. Note that $\delta \tau_{1}=\delta \phi_{1}^{t}=0$ and the mean square errors (MSE), *i.e.*, $\sigma_{\tau }^{2}$, $\sigma_{\phi }^{2}$ and $\sigma_{\varphi }^{2}$ are lower bounded by the CRBs derived in Section 3.

### 4.2. Coherence Performance Analysis

It is obvious from Equation (52) that all the three estimation errors, *i.e.*, *δτ_k_*, $\delta \phi_{k}^{t}$ and *δφ_k_*, will degrade the coherence gain. For convenience, we assume that linear frequency modulation (LFM) signal with a large time-bandwidth product is adopted and the range envelope after pulse compression is modeled as a sinc function:

(53) $$p(t)=\sin\text{c}(\pi Bt),\left|t\right|\leq T_{p}$$

where *B* is the bandwidth of LFM with *β*^2^ = *B*^2^/12 and *T_p_* represents the pulse width.

First we calculate the averaged power of the output signal. The signal in Equation (52) is sampled at *t* = *τ*_1_ where a peak after integration is expected, and the noise-free sampled signal can be expressed as:

(54) $$r(\tau_{1})=\xi \sum_{k=1}^{K}p(\delta \tau_{k})e^{j\delta \phi_{k}^{t}}A(\delta \varphi_{k})$$

where the sinc function *p*(*t*) is applied.

The computation of the averaged power in Equation (54) is provided in Appendix B and the final result is:

(55) $$\begin{array}{ll} P_{SO} & =E\left[{\left|r(\tau_{1})\right|}^{2}\right]\\ & ={\left|\xi \right|}^{2}\left\{\begin{array}{l} \left[1+\left(K-1\right)T_{2}\right]•\left[N+2\sum_{n=1}^{N-1}\left(N-n\right)•e^{-\frac{1}{2}n^{2}\sigma_{\varphi }^{2}}\right]\\ +\left[2\left(K-1\right)T_{1}•e^{-\frac{\sigma_{\phi }^{2}}{2}}+\left(K-1\right)\left(K-2\right)T_{1}^{2}•e^{-\sigma_{\phi }^{2}}\right]•{\left[\sum_{n=0}^{N-1}e^{-\frac{1}{2}n^{2}\sigma_{\varphi }^{2}}\right]}^{2} \end{array}\right\} \end{array}$$

where $T_{1}(\sigma_{\tau }^{2})=E[p(\delta \tau_{2})]$ and $T_{2}(\sigma_{\tau }^{2})=E[{\left|p(\delta \tau_{2})\right|}^{2}]$.Note that $T_{1}(\sigma_{\tau }^{2})$ and $T_{2}(\sigma_{\tau }^{2})$ are both monotone decreasing functions of $\sigma_{\tau }^{2}$ where *T*_1_(0) = *T*_2_(0) = 1 and *T*_1_(∞) = *T*_2_(∞) = 0. When $\sigma_{\tau }^{2}$ is relatively small, e.g., $0\leq \sigma_{\tau }^{2}\leq 0.003$, *T*_1_ and *T*_2_ can be approximated by Taylor expansion of *p*(*t*):

(56) $$\begin{array}{ll} p(t) & =\sin(\pi Bt)/\left(\pi Bt\right)\\ & =\left[(\pi Bt)-\frac{{\left(\pi Bt\right)}^{3}}{3!}+\frac{{\left(\pi Bt\right)}^{5}}{5!}-\cdots \right]/\left(\pi Bt\right)\\ & =1-\frac{{\left(\pi B\right)}^{2}}{6}t^{2}+\frac{{\left(\pi B\right)}^{4}}{120}t^{4}-\cdots \end{array}$$

However, when $\sigma_{\tau }^{2}$ is large, the lower-order Taylor expansion is no longer suitable and polynomial fitting is used to calculate T1 and *T*_2_. In detail, the curves of $T_{1}(\sigma_{\tau }^{2})$ and $T_{2}(\sigma_{\tau }^{2})$ are obtained by Monte-Carlo simulations first and then fitted into two groups of polynomial coefficients. In summary, the analytic formulas of $T_{1}(\sigma_{\tau }^{2})$ and $T_{2}(\sigma_{\tau }^{2})$ are expressed as:

(57) $$T_{1}\left(\sigma_{\tau }^{2}\right)=\{\begin{array}{ll} 1+c_{1}•\sigma_{\tau }^{2}+c_{2}•\sigma_{\tau }^{4}, & 0\leq \sigma_{\tau }^{2}\leq 0.03\\ \text{polyval}\left({\text{P}}_{1},B^{2}\sigma_{\tau }^{2}\right), & 0.03<\sigma_{\tau }^{2}\leq 40 \end{array}$$

and:

(58) $$T_{2}\left(\sigma_{\tau }^{2}\right)=\{\begin{array}{ll} 1+2c_{1}•\sigma_{\tau }^{2}, & 0\leq \sigma_{\tau }^{2}\leq 0.03\\ \text{polyval}\left({\text{P}}_{2},B^{2}\sigma_{\tau }^{2}\right), & 0.03<\sigma_{\tau }^{2}\leq 40 \end{array}$$

where *c*_1_ = −(*πB*)^2^/6, *c*_2_ = (*πB*)^4^/40 and *polyval*(***P***, *x*) represents the value of a polynomial evaluated at *x* with vector ***P*** containing the polynomial coefficients. In the simulations, we use the following two groups of 20-order polynomial coefficients:

(59) $${\text{P}}_{1}=\left[\begin{array}{llllll} 5.8018\text{e}-24 & -2.4016\text{e}-21 & 4.6149\text{e}-19 & -5.4646\text{e}-17 & 4.4633\text{e}-15 & -2.6672\text{e}-13\\ 1.2070\text{e}-11 & -4.2245\text{e}-10 & 1.1577\text{e}-08 & -2.4994\text{e}-07 & 4.2574\text{e}-06 & -5.7041\text{e}-05\\ 5.9680\text{e}-04 & -4.8194\text{e}-03 & 2.9538\text{e}-02 & -1.3430\text{e}-01 & 4.3949\text{e}-01 & -9.9565\text{e}-01\\ 1.4904\text{e}+00 & -1.4167\text{e}+00 & 9.8609\text{e}-01 & & & \end{array}\right]$$

(60) $${\text{P}}_{2}=\left[\begin{array}{llllll} 1.4338\text{e}-23 & -5.8985\text{e}-21 & 1.1259\text{e}-18 & -1.3233\text{e}-16 & 1.0719\text{e}-14 & -6.3475\text{e}-13\\ 2.8435\text{e}-11 & -9.8387\text{e}-10 & 2.6616\text{e}-08 & -5.6629\text{e}-07 & 9.4847\text{e}-06 & -1.2461\text{e}-04\\ 1.2739\text{e}-03 & -1.0004\text{e}-02 & 5.9224\text{e}-02 & -2.5756\text{e}-01 & 7.9401\text{e}-01 & -1.6530\text{e}+00\\ 2.1792\text{e}+00 & -1.7018\text{e}+00 & 9.2949\text{e}-01 & & & \end{array}\right]$$

where the polynomial coefficients are stacked in row-order and descending powers.

From Equation (52), the noise power after integration is $P_{NO}=N\sigma_{w}^{2}$, then the formula of coherence gain for NGR with pulse trains can be expressed as:

(61) $$\begin{array}{ll} G_{\text{NGR}\_\text{MSlave}} & =\frac{SNR_{\text{out}}}{SNR_{in}}=\frac{P_{SO}/P_{NO}}{P_{SI}/P_{NI}}\\ & =\frac{1}{N}\left\{\begin{array}{l} \left[1+\left(K-1\right)T_{2}\right]•\left[N+2\sum_{n=1}^{N-1}\left(N-n\right)•e^{-\frac{1}{2}n^{2}\sigma_{\varphi }^{2}}\right]\\ +\left[2\left(K-1\right)T_{1}•e^{-\frac{\sigma_{\phi }^{2}}{2}}+\left(K-1\right)\left(K-2\right)T_{1}^{2}•e^{-\sigma_{\phi }^{2}}\right]•{\left[\sum_{n=0}^{N-1}e^{-\frac{1}{2}n^{2}\sigma_{\varphi }^{2}}\right]}^{2} \end{array}\right\} \end{array}$$

where $P_{SI}/P_{NI}={\left|\xi \right|}^{2}/\sigma_{w}^{2}$ represents the input SNR.

### 4.3. Remarks on Coherence Performance

Based on Equation (61), we have the following remarks on the coherence performance:

(1) If $\sigma_{\tau }^{2}=\sigma_{\phi }^{2}=\sigma_{\varphi }^{2}=0$, *i.e.*, the estimators are ideally accurate, the maximum SNR gain of *K*^2^*N* can be obtained. Note that *K*^2^ is the ideal SNR gain of a master-slave single-pulse NGR consisting of *K* radars, and *N* is the ideal SNR gain of integrating *N* pulses coherently for a master-slave NGR with a single radar. This concept of joint space-time coherence indicates that more pulses can be employed to exchange for fewer radars to obtain a desired coherence gain, which makes the NGR system more flexible.
(2) If $\sigma_{\tau }^{2}=\sigma_{\phi }^{2}=\sigma_{\varphi }^{2}=\infty$, *i.e.*, the estimation accuracy is extremely low, the minimum SNR gain of 1 or 0 dB can be obtained. The explanations are as follows: $\sigma_{\tau }^{2}=\infty$ means that the echoes emitted from other radars can be hardly aligned with the echo emitted from the master radar, and thus no spatial coherence gain can be obtained. Meanwhile, $\sigma_{\varphi }^{2}=\infty$ means that a random Doppler compensation phase is multiplied to each transmitted pulse of the master radar, so the coherency of the *N* pulses is completely corrupted and the signals are integrated incoherently instead of coherently, which amplifies the power of signal by a factor of *N*. Note that the noise power is also amplified by *N* times, we have the minimum SNR gain of 1.
(3) If we replace $\sigma_{\tau }^{2}$, $\sigma_{\phi }^{2}$ and $\sigma_{\varphi }^{2}$ with the CRBs given in Equations (29), (45) and (46), respectively, then (61) gives an upper bound for the coherence gain of NGR *versus* input SNR.

## 5. Numerical Results

In this section, numerical results are presented and discussions are conducted to verify the CRBs of the GCPs and evaluate the coherence gain formula.

### 5.1. Simulations on CRB

Since the CRBs are not impacted by the number of radars *K*, for simplicity we consider a NGR system with master-slave architecture consisting of two radars. The LFM signal with a time-bandwidth product of 100 is applied in simulation and all the GCPs are estimated by the maximum likelihood estimator (MLE), which is asymptomatically unbiased and efficient, using Monte Carlo simulations with 500 iterations per SNR value.

**Figure 2.**
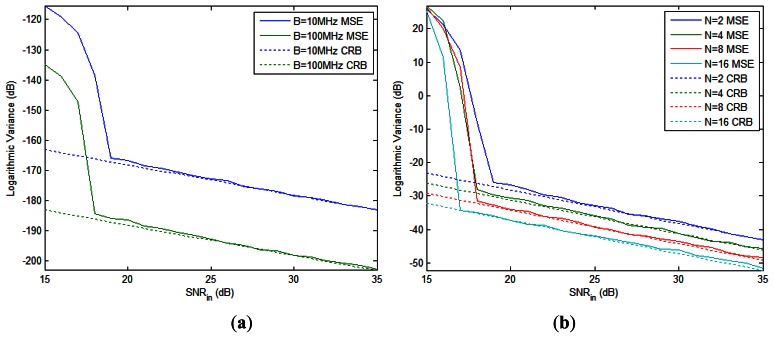
The logarithmic MSE and CRB of (**a**) the time delay difference and (**b**) the normalized time delay difference *vs.* input SNR.

The MSE and CRB of the time delay difference *versus* SNR are shown in Figure 2(a), where *N* = 2 and the bandwidth *B* increases from 10 MHz to 100 MHz. It is seen that larger *B* gives better estimation accuracy. As SNR increases, the MSE descends quickly and approaches the CRB in the high-SNR region. Since the time delay measurement in radar system is usually scaled by the temporal resolution, *i.e.*, 1/*B*, we measure the CRB of the normalized time delay differences which is, from Equation (29), given by:

(62) $$\begin{array}{cc} CRB_{B\Delta \tau_{k}}=\frac{3}{\pi^{2}}\frac{1}{N}\frac{1}{SNR_{in}}, & k=2,\cdots ,K \end{array}$$

Note that the *CRB_B_*_Δ_*_τk_* in Equation (62) is irrelevant to *β* and *B*. Figure 2(b) depicts the MSE and CRB of the normalized time delay difference for *B* = 100 MHz with different number of pulses. We see that the MSE under each *N* value asymptomatically approaches the corresponding CRB curve, and the CRB is decreased by 3 dB when *N* is doubled.

**Figure 3.**
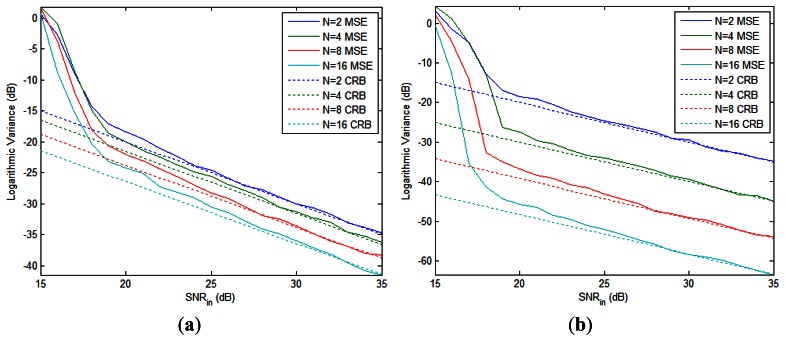
The logarithmic MSE and CRB of (**a**) the total phase difference and (**b**) the Doppler frequency *vs.* input SNR.

The MSE and CRB of the total phase difference and the first Doppler frequency (we have two Doppler frequencies to estimate since *K* = 2) with different number of pulses are shown in Figure 3(a) and (b), respectively. As expected, the MSEs approach the CRBs for high SNR in all cases. The CRB of Doppler frequency declines faster than that of total phase difference when *N* doubles, as we have analyzed in Section 3.4.

### 5.2. Simulations on Coherence Performance

**Figure 4.**
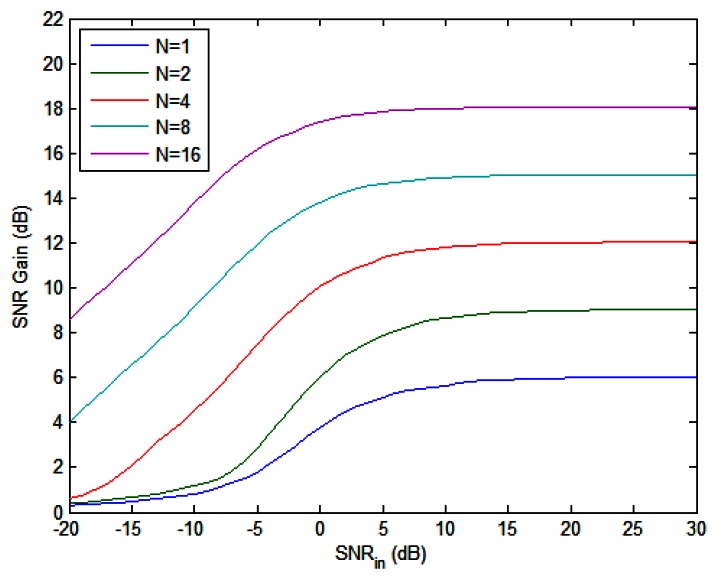
SNR gain *vs.* input SNR with different number of pulses.

The upper bound of SNR gain in Equation (61) *vs.* input SNR is plotted in Figure 4 with different number of pulses when *K* = 2. For a fixed *N*, the SNR gain ascends from 0 dB to a maximum limit value as the input SNR increases. When *N* doubles, the maximum gain increases by 3 dB, *i.e.*, more pulses means better performance. Moreover, the input SNR required to achieve a desired SNR gain can be reduced by applying more pulses if we have a fixed number of radars, as can be seen from Figure 4. Finally, it should be pointed out that the SNR gain curves plotted here only provide a performance bound for NGR, since the practical estimation algorithms cannot reach the CRB in the low-SNR region.

### 5.3. Comparative Simulations

**Figure 5.**
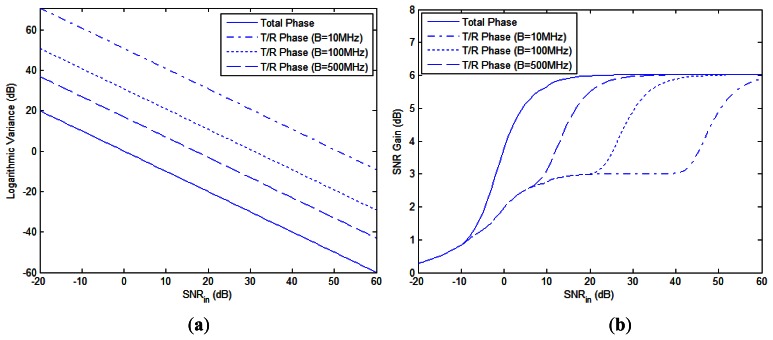
Comparisons between total phase differences and T/R phase differences: (**a**) the logarithmic CRB (**b**) SNR gain *vs.* input SNR.

As mentioned in the second remark of Section 3.4, the CRB of the total phase differences in Equation (45) is irrelevant to *β* and *f*_c_, while the CRB of the T/R phase differences derived in [4] is proportional to (*f*_c_/*β*)^2^. The CRBs of these types of phase errors and the SNR gain based on each of them are shown in Figure 5(a) and (b), respectively, where *N* = 1, *f*_c_ = 1 GHz and the bandwidth *B* increases from 10 MHz to 500 MHz. It is seen that when *B* is relatively small, e.g., *B* = 10 MHz, the CRB of the T/R phase differences is nearly 50 dB higher than that of total phase differences and an input SNR of about 60 dB is required to reach the optimal SNR gain of 6 dB, which makes NGR *seem impractical* with narrowband signal. As mentioned in Section 3.4, performance bound based merely on the T/R phase errors, as done in [4] is inappropriate, for the misalignment of time delay also plays an important role in the performance analysis of NGR. Total phase differences which consist of both the T/R phases and the phase caused by time delay, characterize the signal model more accurately.

## 6. Conclusions

We have extended the NGR model based on a single pulse to the case of pulse trains, so that the coherence gain can be obtained from both spatial and temporal integration. Accordingly, the coherence parameters (CPs) in [2–5] are extended to the generalized coherence parameters (GCPs), with Doppler frequencies involved and the total phase differences introduced. Based on the signal model of NGR with master-slave architecture in MIMO mode, the closed-form CRBs of the GCPs are derived and verified using simulations. Moreover, in order to investigate the impact of estimation errors of GCPs on coherence performance, we developed the signal model of NGR in the coherent mode and derived an analytical bound of SNR gain, which relies on the CRBs of all the GCPs. Our simulations show that the introduction of multiple pulses in NGR not only improves the estimation accuracy and the maximum coherence performance, but also reduces the input SNR required to achieve a desired SNR gain, compared with the case of a single pulse.

## Appendix A

In this Appendix, we develop the FIM for the intermediate parameter vector **θ** defined in Equation (14), based on the log-likelihood function given in Equation (15). It is easy to get the first derivatives of the GCPs as (The primitive function log *p*(**r; θ**) and the integration time  are omitted for simplicity):

(63) ∂∂τk=2σw2Re[ξ∑n=0N−1∫∂(∑k=1Ksk(t−τk)ejΔϕkt+jφk•n)/∂τk•(r1,n(t)−∑k=1Kξsk(t−τk)ejΔϕkt+jφk•n)*dt]

(64) ∂∂Δϕkt=2σw2Re[ξ∑n=0N−1∫∂(∑k=1Ksk(t−τk)ejΔϕkt+jφk•n)/∂ϕkt•(r1,n(t)−∑k=1Kξsk(t−τk)ejΔϕkt+jφk•n)*dt]

(65) ∂∂φk=2σw2Re[ξ∑n=0N−1∫∂(∑k=1Ksk(t−τk)ejΔϕkt+jφk•n)/∂φk•(r1,n(t)−∑k=1Kξsk(t−τk)ejΔϕkt+jφk•n)*dt]

(66) ∂∂ξr=2σw2Re[∑n=0N−1∫(∑k=1Ksk(t−τk)ejΔϕkt+jφk•n)•(r1,n(t)−∑k=1Kξsk(t−τk)ejΔϕkt+jφk•n)*dt]

(67) ∂∂ξi=2σw2Re[j∑n=0N−1∫(∑k=1Ksk(t−τk)ejΔϕkt+jφk•n)•(r1,n(t)−∑k=1Kξsk(t−τk)ejΔϕkt+jφk•n)*dt]

Applying the properties of orthogonal waveforms, we have the second derivatives of the GCPs as:

(68) $$E\left[\frac{\partial^{2}}{\partial \tau_{k}\partial \tau_{l}}\right]=\{\begin{array}{cc} \frac{1}{\sigma_{w}^{2}}{\left|\xi \right|}^{2}8\pi^{2}\beta^{2}N, & k=l\\ 0, & \text{others} \end{array}$$

(69) $$E\left[\frac{\partial^{2}}{\partial \Delta \phi_{k}^{t}\partial \Delta \phi_{l}^{t}}\right]=\{\begin{array}{cc} \frac{2}{\sigma_{w}^{2}}{\left|\xi \right|}^{2}N, & k=l\\ 0, & \text{others} \end{array}$$

(70) $$E\left[\frac{\partial^{2}}{\partial \varphi_{k}\partial \varphi_{l}}\right]=\{\begin{array}{ll} \frac{1}{\sigma_{w}^{2}}{\left|\xi \right|}^{2}\frac{N\left(N-1\right)\left(2N-1\right)}{3}, & k=l\\ 0, & \text{others} \end{array}$$

(71) $$E\left[\frac{\partial^{2}}{\partial {\left(\xi_{r}\right)}^{2}}\right]=E\left[\frac{\partial^{2}}{\partial {\left(\xi_{i}\right)}^{2}}\right]=\frac{2}{\sigma_{w}^{2}}KN$$

(72) $$E\left[\frac{\partial^{2}}{\partial \tau_{k}\partial \Delta \phi_{l}^{t}}\right]=E\left[\frac{\partial^{2}}{\partial \tau_{k}\partial \varphi_{l}}\right]=E\left[\frac{\partial^{2}}{\partial \tau_{k}\partial \xi_{r}}\right]=E\left[\frac{\partial^{2}}{\partial \tau_{k}\partial \xi_{i}}\right]=E\left[\frac{\partial^{2}}{\partial \xi_{r}\partial \xi_{i}}\right]=0$$

(73) $$E\left[\frac{\partial^{2}}{\partial \Delta \phi_{k}^{t}\partial \varphi_{l}}\right]=\{\begin{array}{cc} \frac{1}{\sigma_{w}^{2}}{\left|\xi \right|}^{2}N\left(N-1\right), & l\neq 1\text{and k}=l\\ 0, & \text{others} \end{array}$$

(74) $$E\left[\frac{\partial^{2}}{\partial \Delta \phi_{k}^{t}\partial \xi_{r}}\right]=-\frac{2}{\sigma_{w}^{2}}N\xi_{i}$$

(75) $$E\left[\frac{\partial^{2}}{\partial \Delta \phi_{k}^{t}\partial \xi_{i}}\right]=\frac{2}{\sigma_{w}^{2}}N\xi_{r}$$

(76) $$E\left[\frac{\partial^{2}}{\partial \varphi_{k}\partial \xi_{r}}\right]=-\frac{1}{\sigma_{w}^{2}}N\left(N-1\right)\xi_{i}$$

(77) $$E\left[\frac{\partial^{2}}{\partial \varphi_{k}\partial \xi_{i}}\right]=\frac{1}{\sigma_{w}^{2}}N\left(N-1\right)\xi_{r}$$

From Equations (68) to (77), the FIM defined in Equation (16) is obtained.

## Appendix B

In this Appendix, we develop the averaged power of the output signal. From Equation (54), we have:

(78) $$\begin{array}{ll} P_{SO} & =E\left[{\left|r(\tau_{1})\right|}^{2}\right]\\ & =E\left[r(\tau_{1})r^{*}(\tau_{1})\right]\\ & ={\left|\xi \right|}^{2}E\left\{\left[\sum_{k=1}^{K}p(\delta \tau_{k})e^{j\delta \phi_{k}^{t}}A(\delta \varphi_{k})\right]•\left[\sum_{l=1}^{K}p(\delta \tau_{l})e^{-j\delta \phi_{l}^{t}}A^{*}(\delta \varphi_{l})\right]\right\}\\ & ={\left|\xi \right|}^{2}E\left[\sum_{k=1}^{K}\sum_{l=1}^{K}p(\delta \tau_{k})p(\delta \tau_{l})e^{j\delta \phi_{k}^{t}-j\delta \phi_{l}^{t}}A(\delta \varphi_{k})A^{*}(\delta \varphi_{l})\right] \end{array}$$

The i.i.d. property of the estimation errors and the following equations will be applied hereinafter: $\delta \tau_{1}=\delta \phi_{1}^{t}=0$, *E* [*A*(δφ_k_)]=*E* [*A**(δφ_k_)], $E\left[e^{\pm j\delta \phi_{k}^{t}}\right]=e^{-\frac{1}{2}\sigma_{\phi }^{2}}$ and $E\left[e^{\pm j\delta \varphi_{k}}\right]=e^{-\frac{1}{2}\sigma_{\varphi }^{2}}$. Then we calculate the sum in Equation (78) in the following three cases:

(1) *k* = *l*
  This part of the sum can be expressed as:
  (79) $$\begin{array}{ll} P_{1} & =E\left\{\left[A(\delta \varphi_{1})A^{*}(\delta \varphi_{1})+\sum_{k=2}^{K}{\left|p(\delta \tau_{k})\right|}^{2}A(\delta \varphi_{k})A^{*}(\delta \varphi_{k})\right]\right\}\\ & =E\left[A(\delta \varphi_{1})A^{*}(\delta \varphi_{1})\right]+\sum_{k=2}^{K}E\left[{\left|p(\delta \tau_{k})\right|}^{2}\right]E\left[A(\delta \varphi_{k})A^{*}(\delta \varphi_{k})\right]\\ & =\left\{1+\left(K-1\right)E\left[{\left|p(\delta \tau_{2})\right|}^{2}\right]\right\}•E\left[{\left|A(\delta \varphi_{1})\right|}^{2}\right]\\ & =\left[1+\left(K-1\right)T_{2}\right]•E\left[{\left|A(\delta \varphi_{1})\right|}^{2}\right] \end{array}$$
(2) *k* = 1, *l* = 2, …, *K* or *l* = 1, *k* = 2, …, *K*
  This part of the sum can be expressed as:
  (80) $$\begin{array}{ll} P_{2} & =E\left[A^{*}(\delta \varphi_{1})•\sum_{k=2}^{K}p(\delta \tau_{k})e^{j\delta \phi_{k}^{t}}A(\delta \varphi_{k})+A(\delta \varphi_{1})•\sum_{l=2}^{K}p(\delta \tau_{l})e^{-j\delta \phi_{l}^{t}}A^{*}(\delta \varphi_{l})\right]\\ & =2E\left[A^{*}(\delta \varphi_{1})•\sum_{k=2}^{K}p(\delta \tau_{k})e^{j\delta \phi_{k}^{t}}A(\delta \varphi_{k})\right]\\ & =2\left(K-1\right)•E\left[p(\delta \tau_{2})\right]•E\left[e^{j\delta \phi_{2}^{t}}\right]•{\left|E\left[A(\delta \varphi_{1})\right]\right|}^{2}\\ & =2\left(K-1\right)T_{1}•e^{-\frac{\sigma_{\phi }^{2}}{2}}•{\left|E\left[A(\delta \varphi_{1})\right]\right|}^{2} \end{array}$$
(3) *k* ≠ *l*, and *k*, *l ≥* 2
  This part of the sum can be expressed as:
  (81) $$\begin{array}{ll} P_{3} & =E\left[\sum_{k=2}^{K}\sum_{l=2,k\neq l}^{K}p(\delta \tau_{l})p(\delta \tau_{k})e^{j\delta \phi_{k}^{t}}e^{-j\delta \phi_{l}^{t}}A(\delta \varphi_{k})A^{*}(\delta \varphi_{l})\right]\\ & =\left(K-1\right)\left(K-2\right)E\left[p(\delta \tau_{l})p(\delta \tau_{k})e^{j\delta \phi_{k}^{t}}e^{-j\delta \phi_{l}^{t}}A(\delta \varphi_{k})A^{*}(\delta \varphi_{l})\right]\\ & =\left(K-1\right)\left(K-2\right)•{\left|E\left[p(\delta \tau_{2})\right]\right|}^{2}•{\left|E\left[e^{j\delta \phi_{2}^{t}}\right]\right|}^{2}•{\left|E\left[A(\delta \varphi_{1})\right]\right|}^{2}\\ & =\left(K-1\right)\left(K-2\right)T_{1}^{2}•e^{-\sigma_{\phi }^{2}}•{\left|E\left[A(\delta \varphi_{1})\right]\right|}^{2} \end{array}$$
  In addition, we have:
  (82) $$\begin{array}{ll} E\left[{\left|A(\delta \varphi_{1})\right|}^{2}\right] & =E\left[\sum_{n=0}^{N-1}e^{j\delta \varphi_{1}•n}\sum_{m=0}^{N-1}e^{-j\delta \varphi_{1}•m}\right]\\ & =E\left[N+\sum_{n=0}^{N-1}\sum_{m=0,m\neq n}^{N-1}e^{j\delta \varphi_{1}•\left(n-m\right)}\right]\\ & =N+2\sum_{n=1}^{N-1}\left(N-n\right)•e^{-\frac{1}{2}n^{2}\sigma_{\varphi }^{2}} \end{array}$$
  and:
  (83) $${\left|E\left[A(\delta \varphi_{1})\right]\right|}^{2}={\left|E\left[\sum_{n=0}^{N-1}e^{j\delta \varphi_{1}•n}\right]\right|}^{2}={\left[\sum_{n=0}^{N-1}e^{-\frac{1}{2}n^{2}\sigma_{\varphi }^{2}}\right]}^{2}$$
  From Equations (79) to (83), Equation (78) can be simplified as:
  (84) $$\begin{array}{ll} P_{SO} & ={\left|\xi \right|}^{2}\left(P_{1}+P_{2}+P_{3}\right)\\ & ={\left|\xi \right|}^{2}\left\{\begin{array}{l} \left[1+\left(K-1\right)T_{2}\right]•\left[N+2\sum_{n=1}^{N-1}\left(N-n\right)•e^{-\frac{1}{2}n^{2}\sigma_{\varphi }^{2}}\right]\\ +\left[2\left(K-1\right)T_{1}•e^{-\frac{\sigma_{\phi }^{2}}{2}}+\left(K-1\right)\left(K-2\right)T_{1}^{2}•e^{-\sigma_{\phi }^{2}}\right]•{\left[\sum_{n=0}^{N-1}e^{-\frac{1}{2}n^{2}\sigma_{\varphi }^{2}}\right]}^{2} \end{array}\right\} \end{array}$$
